# Selection for Cell Yield Does Not Reduce Overflow Metabolism in *Escherichia coli*

**DOI:** 10.1093/molbev/msab345

**Published:** 2021-12-06

**Authors:** Iraes Rabbers, Willi Gottstein, Adam M Feist, Bas Teusink, Frank J Bruggeman, Herwig Bachmann

**Affiliations:** 1 Systems Biology Lab, Amsterdam Institute of Molecular and Life Sciences (AIMMS), Vrije Universiteit Amsterdam, Amsterdam, The Netherlands; 2 Department of Bioengineering, University of California San Diego, La Jolla, California, USA; 3 Novo Nordisk Foundation Center for Biosustainability, Technical University of Denmark, Lyngby, Denmark; 4 NIZO Food Research, Ede, The Netherlands

**Keywords:** overflow metabolism, r/k selection, yield, emulsion culturing, metabolic strategy, cell size, experimental evolution

## Abstract

Overflow metabolism is ubiquitous in nature, and it is often considered inefficient because it leads to a relatively low biomass yield per consumed carbon. This metabolic strategy has been described as advantageous because it supports high growth rates during nutrient competition.

Here, we experimentally evolved bacteria without nutrient competition by repeatedly growing and mixing millions of parallel batch cultures of *Escherichia coli*. Each culture originated from a water-in-oil emulsion droplet seeded with a single cell. Unexpectedly we found that overflow metabolism (acetate production) did not change. Instead, the numerical cell yield during the consumption of the accumulated acetate increased as a consequence of a reduction in cell size. Our experiments and a mathematical model show that fast growth and overflow metabolism, followed by the consumption of the overflow metabolite, can lead to a higher numerical cell yield and therefore a higher fitness compared with full respiration of the substrate. This provides an evolutionary scenario where overflow metabolism can be favorable even in the absence of nutrient competition.

## Introduction

**Fig. 1.**
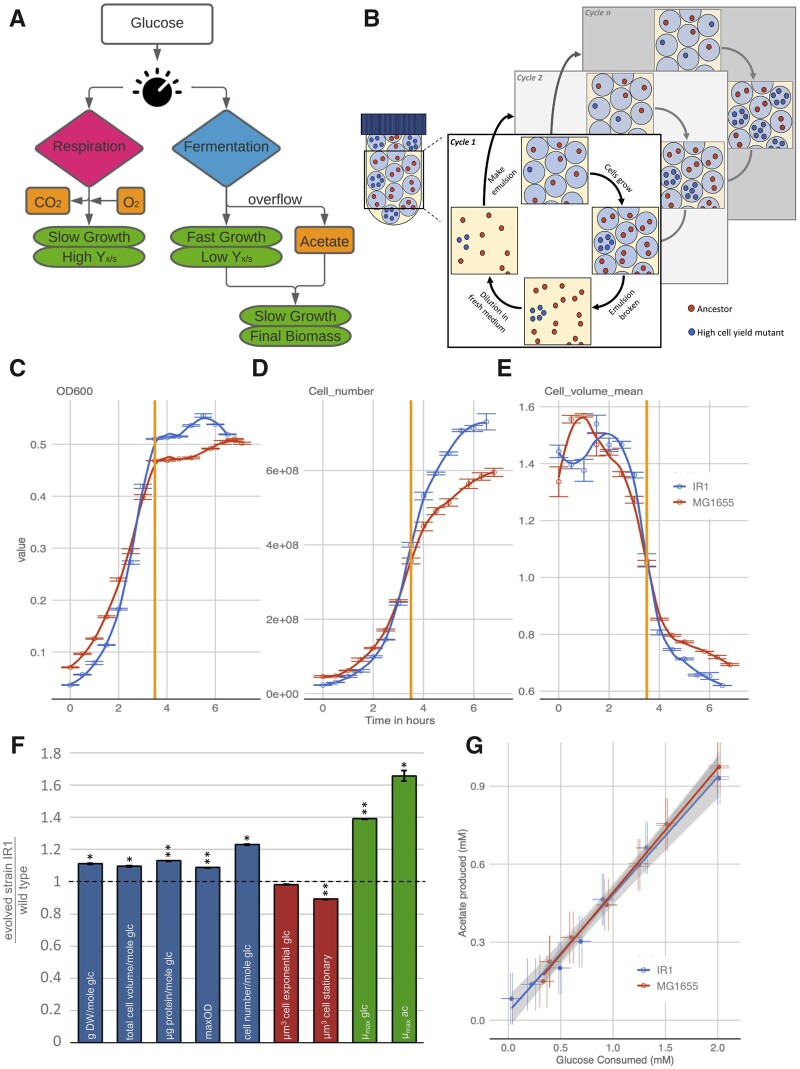
Cell propagation in emulsion droplets resulted in a mutant strain with increased cell yield, but not in a reduction of the acetate production per consumed glucose. (*A*) High biomass yield (Y_x/s_) is commonly associated with respiration. Upon depletion of the primary carbon source, growth on overflow metabolites leads to additional biomass formation. (*B*) Serial propagation of cells grown in emulsion droplets selects for mutants with an increased number of viable offspring per mole glucose (numerical cell yield). Cells were propagated for 53 serial transfers in emulsion and 90 single colonies were isolated and analyzed for their growth rates and final optical densities. (*C*–*E*) In batch culture fast growth on glucose (depletion is indicated with the vertical orange line) is followed by slow growth on the formed acetate. During growth on acetate the cell number (counts/ml) increases much more than the OD. Upon glucose depletion the cell volume (µm^3^) decreases . The effect of cell number/volume is bigger for the evolved strain IR1 compared with the wild-type MG1655. (*F*) A comparison of evolved strain IR1 normalized to the wild-type revealed significant increases in different biomass and cell yield measures (blue), as well as growth rate (green), while the cell size (red) decreased significantly during the second growth phase on acetate. Error bars represent standard deviations, one star indicates two-sided *t-*test *P* value <0.05, two stars *t*-test *P* value <0.005, *n* = 3. For more details on the underlying data, see supplementary table S1 and supplementary figure S2, Supplementary Material online. (*G*) The moles of acetate produced per moles of consumed glucose did not change significantly between the wild-type and the selected strain IR1 (ANOVA; *F* = 0.58, *F*-crit = 4.26; *P* value = 0.45, *α* = 0.05, *n* = 3).

When microbes compete, fast-growing strategies typically become dominant. While numerous microorganisms have the ability to respire during fast growth on high-quality carbon sources like glucose, many bacteria, yeasts, and mammalian cells are known to not make full use of this capability, even when sufficient oxygen is available (Van Hoek et al. 1998; Xu et al. 1999; Jasmin et al. 2012; LaCroix et al. 2015). Instead of efficiently catabolizing the available carbon source to CO_2_, they produce metabolic by-products like acetate, lactate, or ethanol. This phenomenon is termed overflow metabolism (fig. 1*A*). It has been suggested to be evolutionarily favorable at nutrient excess, because it supports the highest growth rates and it allows microorganisms that employ the fermentative/overflow strategy to outcompete fully respiratory cells (Thomas et al. 1979; Molenaar et al. 2009; Bachmann et al. 2013). Overflow metabolism is often regarded as “wasteful” because it reduces the maximum attainable ATP yield of carbon sources (Basan et al. 2015). The reduction in ATP yield at high metabolic rates is thought to be caused by thermodynamical principles. With a higher fraction of the free energy going into ATP production (respiration), the energy available for driving a metabolic pathway, and thereby the growth rate, decreases (Pfeiffer et al. 2001; MacLean 2008; Rabbers et al. 2015).

Contrary to nutrient excess conditions where overflow occurs, cells subjected to low substrate concentrations grow at a lower rate and display full respiratory behavior, for example, in chemostats, which is associated with an increase in metabolic efficiency (mole ATP/mole carbon source or gram biomass/mole carbon source; Garrigues et al. 1997; Jasmin et al. 2012; Bachmann et al. 2013; LaCroix et al. 2015).

In nature, many microbial species are in spatially structured environments where resources can be localized and the microbes undergo cycles of feast and famine. Their metabolic strategies have adapted to those conditions. Characteristic of such conditions are periods of nutrient excess followed by starvation, similar to batch cultures that are continued until complete depletion of the carbon sources. The winning strategy generates the highest number of viable offspring after a single cycle of feast and famine. Thus, microorganisms are faced with an “optimization problem”: the maximal number of viable offspring has to be produced in the shortest possible time given the available resources. In this context, metabolic inefficiency of overflow metabolism is generally considered to be the price for being fast.

As (growth) rate selection is associated with the occurrence of overflow metabolism, one would assume that yield selection should result in an increased metabolic efficiency, typically via enhanced respiratory metabolism. One way to ensure that growth rate competition between microorganisms is prevented, and selection of yield becomes possible, is by culturing individual cells in droplets of water-in-oil emulsions (fig. 1*B*). In this regime each cell gets its own “privatized” amount of substrate within a medium droplet, and does not compete for it with other genotypes, ruling out rate selection. The cells in a droplet can be allowed to grow until depletion of all carbon sources. When *Lactococcuslactis* was subjected to this protocol, the mutant cells that fixed had shifted their metabolism from inefficient homolactic to the more efficient mixed-acid fermentation. These mutants had a higher biomass yield and numerical cell yield at a reduced growth rate (Bachmann et al. 2013). This was the first experimental illustration of (cell) yield selection.

We exploited this emulsion protocol to evolve *Escherichiacoli* MG1655. Unexpectedly we found no reduction of overflow metabolism after experimental evolution, but rather a decrease in cell size, specifically during growth on the overflow metabolite acetate. This led to an increased numerical cell yield (number of cells/mole glucose). Our experiments and a mathematical model show that in a feast–famine regime fast growth and overflow metabolism followed by the consumption of the overflow metabolite can lead to a higher fitness compared with full respiration of the substrate.

## Results

### Selection for Cell Yield Does Not Decrease Overflow Metabolism

The serial propagation of individual microbial cells in emulsion droplets resembles millions of parallel batch cultivations, each inoculated by a single cell. In each droplet, cells can grow for a limited number of generations (in our case 5–6, set by the average droplet size and the available carbon source) before they reach stationary phase. After such a growth period, all droplets are merged, the cells are mixed and diluted, and used to inoculate new droplets for the next round of growth. This protocol (fig. 1*B*) selects for increased numerical cell yield per mole glucose. We wondered if this selection protocol would lead to *E. coli* strains with an increased metabolic efficiency (i.e., yield of gram biomass/mole glucose) and reduced overflow metabolism (more respiratory). To investigate this, we propagated *E. coli* MG1655 for 53 transfers in emulsions and determined the growth characteristics of 90 isolates (supplementary fig. S1, Supplementary Material online). Many of these isolates showed an increased maximal optical density (OD) compared with the wild-type, of which five were further characterized. One strain, designated IR1, was taken for detailed characterization.

Besides the increase in four biomass measures (dry weight, total cell volume, total protein content, and OD) of 9–13%, strain IR1 also showed an increased growth rate on glucose (39% higher) (fig. 1*C*–*F*). Genome sequencing of strain IR1 revealed five mutations compared with its ancestor MG1655 (supplementary table S3, Supplementary Material online). Three of these mutations concerned an uncharacterized phage protein, a guanidine deaminase and a putative nonaryl sulfatase. For those three genes, we were not able to identify a clear link to the observed phenotype. Another mutation concerns an 82 bp deletion in the *rph/pyrE* region that was previously characterized (Conrad et al. 2009; LaCroix et al. 2015). This mutation alleviates a pyrimidine production deficiency of the ancestral strain (Yates and Pardee 1956; Jensen 1993; Valgepea et al. 2011) that limits growth on minimal medium, but not on rich medium. Growth experiments confirmed that IR1 also displays this phenotype (supplementary fig. S3, Supplementary Material online). We also found that during the glucose-growth phase, IR1 shows a 98% decrease in the production of pyrimidine intermediates orotate and dihydroorotate (supplementary fig. S2, Supplementary Material online). Together this data indicated that the increase in yield and rate is partially due to mutations that relieve the pyrimidine deficiency of the ancestral strain. Resequencing further revealed a mutation that leads to a stop codon in the *ygeR* gene (supplementary table S3, Supplementary Material online). This gene is involved in septum formation and cell division, and deletion of it has been shown to reduce cell length (Uehara et al. 2009).

Mutants that showed a shift from fermentation toward respiration were not found. The amount of acetate produced per mole of consumed glucose was not altered significantly in the evolved mutant IR1 (fig. 1*G*). In an additional attempt to isolate mutants with altered overflow metabolism activity, we propagated strain IR1 for another 25 transfers in emulsion droplets. The subsequent characterization of 15 evolved strains showed that all strains still produced acetate (see supplementary table S2, Supplementary Material online).

Another phenotypic change of the isolated mutant IR1, was a decrease in cell volume of 11% compared with the ancestral strain. This cell size decrease was especially obvious during the second growth phase on acetate, and it corresponds to an increase in cell number of 23% (fig. 1*D* and *E*) when the glucose is depleted. This phenotypic change might be related to the mutation found in *ygeR*, which is implicated in cell size reduction (Uehara et al. 2009). The fact that we isolated mutant strains with reduced cell size and that we were not able to identify strains with decreased overflow metabolism led to an alternative hypothesis: In a feast–famine environment where the evolutionary pressure is on the overall numerical cell yield, overflow metabolism is actually more efficient than complete respiration when the consumption of the overflow product acetate is taken into the equation.

### Biphasic Substrate Utilization Optimizes Fitness in Feast–Famine Environments

During growth in batch culture until complete exhaustion of all the carbon sources, microbes are subjected to continuously changing conditions. This resembles a feast and famine cycle with two feast phases (a first phase on glucose and a second phase on acetate), followed by a carbon source exhaustion/famine phase. If the carbon source is a fast fermentable sugar such as glucose then the first phase will be characterized by a high growth rate and a relatively large cell size (Si et al. 2017). As long as the sugar concentration is high, maximizing the growth rate leads to the highest number of offspring produced per unit time. At this stage, it is not relevant if the cell is metabolically inefficient, as there is sufficient substrate available. However, when the initial “fast” substrate becomes limiting, cells will switch to growth on the produced overflow metabolite, acetate in the case of *E. coli*. Growth on an overflow metabolite is always slower than on the initial fast substrate and slow growth is correlated with smaller cell sizes (Schaechter et al. 1958; DeLong et al. 2010; Volkmer and Heinemann 2011; Taheri-Araghi et al. 2015; Si et al. 2017).

We show that already during the onset of the second growth phase, the cells started to reduce in size to eventually reach a cell volume in stationary phase that is less than half of what it was during exponential growth on glucose (fig. 1*E*). During this cell size adaptation period, making new offspring cells costs less nutrients than during steady-state growth on acetate, because a mother cell is now larger than a daughter cell and likely does not need to double in size before division. Immediately after glucose depletion, cells, therefore, need to produce proportionally less biomass to divide into two daughter cells. This leads to a proportionally higher increase in cell number compared with the increase in biomass during the transition from glucose to acetate (fig. 1*C* and *D*). This reduction in cell size during the acetate growth phase is substantially larger compared with conditions where no biphasic growth was possible. We tested this by performing batch culture experiments where growth arrest occurred due to nitrogen limitation rather than due to carbon/acetate depletion (see supplementary information 1, Supplementary Material online). Together, the large differences in cell size on glucose and acetate, the identification of mutants with a smaller cells size, and the consequences of this phenotype on the numerical cell yield suggest significant fitness effects of cell size in feast–famine conditions.

### Rate and Cell Yield Selection Effects of Biphasic Carbon Source Utilization

**Fig. 2.**
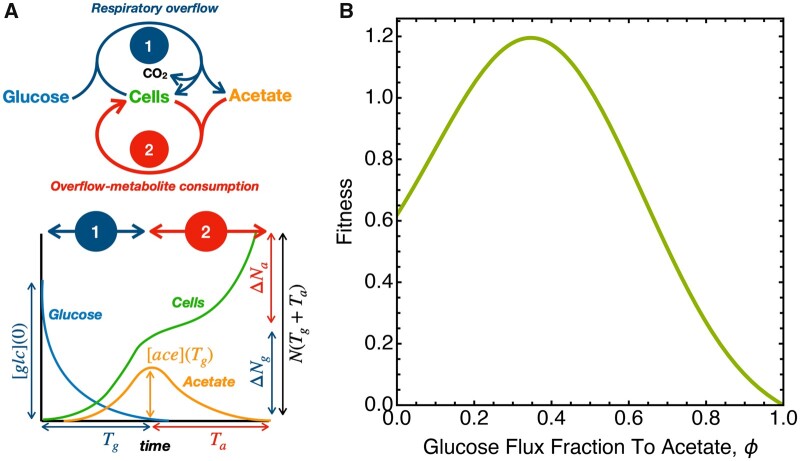
Modeling the fitness consequences of biphasic growth for *E. coli*. (*A*) Schematic overview of metabolic processes associated with biphasic growth: glucose consumption, acetate formation, and growth during the first phase and acetate consumption and growth during the second phase. (*B*) An illustration of the concentrations and cell abundance as function of time during biphasic growth and introduction of terminology. (*C*) We used the model to calculate the fitness (*F*, see Materials and Methods) as function of the fraction of the glucose flux directed to acetate formation. Fitness is defined as the logarithm of the factor increase in the number of cells divided by the time until carbon source depletion, that is, 1/(*T*_g_ + *T*_a_)ln *N*(*T*_a_ + *T*_g_)/*N*(0), in agreement with classical evolutionary biology. Parameters values can be found in Table 1 of the Materials and Methods section.

The widespread interpretation of the evolutionary benefit of overflow metabolism is biased toward the rate selection argument. However, repeated cycles of growth with finite nutrient supply broadly occur in nature, and it is likely to have played an important role during the evolution of metabolic strategies. To address whether biphasic substrate utilization is an evolutionary advantageous strategy to maximize the number of offspring in environments with finite resources, we made a mathematical model (see Materials and Methods for full derivation and description). The model calculates the number of cells that are made from an initial amount of glucose, given the known biomass yields on glucose and acetate, the fraction ϕ of glucose converted into acetate, the cell sizes on glucose and acetate, and the growth rates on glucose and acetate. In agreement with experiments, the growth rate on glucose is a function of ϕ: during pure respiratory growth (at ϕ=0) the growth rate is lower than during overflow metabolism (intermediate ϕ) and when only acetate would be made from glucose (ϕ=1), the growth rate on glucose is zero. A schematic of the model is shown in figure 2*A* and *B*. We used the model to determine the fitness in the case of selection under feast and famine cycles.

Figure  2*C* shows that the qualitative model predicts maximal fitness not when cells fully respire (ϕ = 0) but rather when a fraction of the consumed carbon is first converted to acetate. A fully respiratory cell has a lower fitness than this strategy, indicating that selection during feast–famine conditions selects for a phenotype that combines fast growth (overflow metabolism on glucose) with small cell/high cell number (growth on acetate).

## Discussion

Studies describing overflow metabolism as inefficient typically do not include the consumption of overflow metabolites when comparing it to yields of fully respiratory cells. This reasoning is valid during constant conditions of high glucose concentrations where overflow metabolism indeed reduces the maximum attainable ATP (and gram biomass) yield of the utilized carbon source (Postma et al. 1989; Pfeiffer and Bonhoeffer 2006; MacLean 2008; Molenaar et al. 2009). It does, however, not apply when the carbon source is depleted, for example, in batch cultures when utilization of a carbon source is followed by the consumption of the overflow metabolites. Therefore, for a fair comparison, the additional biomass yield on the overflow metabolites needs to be considered as well. In this case it is not immediately clear whether fully respiratory growth is a better evolutionary strategy than overflow metabolism and biphasic growth.

Interestingly, many bacteria become larger when they grow faster; also *E. coli* is known to have a positive correlation between growth rate and cell size (Schaechter et al. 1958; DeLong et al. 2010; Volkmer and Heinemann 2011; Taheri-Araghi et al. 2015). This occurs when the generation time is shorter than the DNA replication time, necessitating mother cells to start DNA replication for their future progeny (Donachie 1968; Skarstad et al. 1986; Bipatnath et al. 1998). During evolution on a finite nutrient supply, however, it pays off to make a higher number of cells per unit nutrient, and this can be achieved by making smaller cells. A fast growth rate that supports an evolutionary advantage under nutrient excess, therefore has a trade-off with a lower number of offspring per unit of substrate. This trade-off is driven by cell size and independent of metabolic efficiency. An optimization problem thus occurs, where small (but slow) phenotypes would generally be outgrown by faster growing cells during nutrient competition. We circumvented this trade-off by serially propagating cells in emulsion droplets where single cells are allowed to grow until nutrient exhaustion in physically separated environments. In this regime mutants that produce more offspring will seed more droplets in the next round and increase in frequency. As such a selection in emulsion led to increased metabolic efficiency, biomass- and cell-yield in *L. lactis* (Bachmann et al. 2013), we anticipated that this protocol might allow to reduce overflow metabolism in *E. coli*.

Against expectations, our experiments did not show that full respiration was a competitive strategy when rate selection was excluded. We found that in *E. coli* the utilization of overflow metabolism was unaffected. However, we did select for a mutant with a smaller cell size, which is possibly linked to a single base deletion in *ygeR*, a gene known to affect the cell size (Uehara et al. 2009). We identified two reasons for the difference in the outcome between the *L. lactis* study and our experiments 1) *E. coli* is able to consume the overflow metabolite which *L. lactis* cannot, and 2) when growing on the overflow metabolite, the cell number of *E. coli* increases disproportionately compared with the biomass increase.

Our results show that overflow metabolite production and its subsequent utilization maximize the number of produced offspring irrespective of the available substrate concentration. At high substrate concentrations the growth rate will be maximized while at limiting substrate concentrations a minimization of cell size (maximization of cell number) will occur after the initial “fast” substrate is depleted. This allows the optimization of fitness in dynamic, for example, feast-famine environments. Maximizing the production rate of offspring through sequential substrate utilization seems a very elegant solution that might apply to numerous organisms. For instance, the secretion of ethanol, lactate, or acetate and their subsequent consumption are broadly encountered (El-Mansi and Holms 1989; Goffin et al. 2004; Thomson et al. 2005). Like for *E. coli*, other organisms showing such behavior are also described to have a positive correlation between growth rate and cell size (Johnston et al. 1979; Bachmann et al. 2013; Taheri-Araghi et al. 2015). This makes it plausible that a similar fitness maximization strategy in dynamic environments might apply to a broad range of organisms.

Attempts to engineer Crabtree negative yeast and *E. coli* strains that do not display overflow metabolism, have been only partially successful and typically the obtained strains are growing poorly or showing unexpected metabolic activities (De Mey et al. 2007). This points to overflow metabolism being a robust property of the metabolic networks that is difficult to overcome. However, Crabtree negative yeasts are known and it would be interesting to understand under which conditions they might have evolved (Hagman et al. 2013; Pfeiffer and Morley 2014). As the selective pressure we applied is on the numerical cell yield, one could also imagine that once a minimum cell size is reached the only way to further increase the number of cells is by shifting to full respiration. Our attempts to reach this by further propagating IR1 in emulsion did not lead to such a shift, but that could be due to the limited number of transfers applied. We cannot exclude that an alternative strategy exists that would lead to smaller and fully respiratory cells.

There are numerous computational approaches that predict the shift from respiration toward overflow metabolism, based on biochemical and biophysical constraints. These studies include, for example, flux balance analysis, where the optimal metabolic flux distribution is predicted that supports high growth rates, trade-offs due to membrane crowding, optimization of proteome allocation to minimize the investment associated with the costly respiratory machinery, and thermodynamic control of acetate production through the extracellular acetate concentration (Ibarra et al. 2002; Müller et al. 2014; Wortel et al. 2014; Basan et al. 2015; Nilsson and Nielsen 2016; Enjalbert et al. 2017; Long et al. 2017; Szenk et al. 2017; Millard et al. 2021). However, besides some studies in yeast where the main argument is that overflow metabolites lead to a fitness advantage due to their toxicity for competing organisms (Make–Accumulate–Consume hypothesis) (Thomson et al. 2005; Piškur et al. 2006; Hagman et al. 2013) the role of evolutionary perspectives are less investigated. The discussion of overflow metabolism in literature and textbooks sometimes neglects the fact that the secreted overflow metabolite is often further metabolized. Our results argue for the consideration of the consumption of the overflow metabolite as it likely played a role during evolution in natural environments. This is corroborated by a recent study on the regulation of overflow metabolism (Millard et al. 2021). Full respiration and reaching the maximal biomass yield are only seen at low substrate concentrations. This might be a rather artificial situation which is created for instance in chemostats. In nature, cells are often exposed to dynamic feast–famine cycles, and spatial structure that leads to variations in the selection pressure.

If rate selection would be the only force to favor overflow metabolism one might expect higher acetate fractions to be produced by the wild-type strain. The fact that in most cases only a small fraction of the flux is directed toward acetate argues for other (additional) selective forces, such as maximizing cell number. In studies by LaCroix et al (2015) and Long et al. (2017), rate selection was applied to the same *E. coli* MG1655 wild-type strain. They found a positive correlation between the acetate production and growth rates in the wild-type and evolved strains. Such an increase in overflow metabolism at increasing growth rates has been reported in a number of other studies as well (Thomas et al. 1979; Van Hoek et al. 1998; Kayser et al. 2005; Valgepea et al. 2010). This might indicate that the natural environment from which the wild-type strain was isolated was not purely selective for growth rate.

Another aspect of natural environments is the possibility of multiple species or strains coexisting. A “cheater” strain that consumes the acetate before the producer strain can benefit from it would potentially argue against a beneficial effect of overflow metabolism beyond growth rate maximization (Maclean and Gudelj 2006). However, growth on acetate is typically slow, so even if such a strain would be present at high frequency in the population, its consumption rate would still be significantly lower than what is needed to fully deplete this substrate before the fast growing/acetate producing biomass starts consuming it. In order to pose significant competition, the acetate consumer would therefore need to grow at a similar rate as the acetate producer. Almost all growth rates we found for diverse organisms on acetate were substantially lower than this (see supplementary table S5, Supplementary Material online for a number of examples of glucose and acetate growth rate ratios), except for an *Acinetobacter* strain that displays an exceptionally high growth rate on acetate of 0.91 h^−1^ (Sigala et al. 2019).

In conclusion, this study shows an unexplored consequence of overflow metabolism. It is not only a strategy for fast growth at excess substrate conditions, but it also maximizes the number of offspring in dynamic conditions with regular episodes of finite substrate concentrations. From an evolutionary perspective overflow metabolism is potentially selected because it maximizes the number of offspring in such dynamic environments, be it through a fast growth rate or the minimization of the cell size. Our results argue for a revisited perspective when investigating metabolic strategies, as currently consumption of overflow metabolites and the role of cell numbers during selection are neglected when fermentation is compared with full respiration.

## Materials and Methods

### Strains and Media

For experimental evolution *E.coli* K12 substr. MG1655 (isolate BOP27 obtained from Palsson lab at UCSD) was used. The experiments were initiated from a single colony glycerol stock which was cultured in three biological replicates. Strains were cultured at 37 °C using M9 minimal medium (LaCroix et al. 2015) supplemented with 2.5 mM glucose. The same medium was used throughout this study.

### Culturing in Emulsion

A fully-grown cell culture of *E. coli* was diluted to a concentration of ∼2 × 10^6^ cells ml^−1^ in M9 medium supplemented with 2.5 mM glucose. Using 700 µl of the diluted cell suspension and 300 µl Novec HFE 7500 fluorinated oil (3 M, Maplewood, MN) containing 0.1% Pico-Surf™ surfactant (Dolomite Microfluidics, Hertfordshire, UK) emulsions were prepared as described earlier (Bachmann et al. 2013). In such an emulsion ∼1 in 10 droplets (∼50 µm in diameter) will be inoculated with a single cell (following a Poisson distribution). After overnight incubation at 37 °C, the emulsions were broken using perfluoroctanol (Alfa Aesar Haverhill, MA) and the cultures were diluted in fresh medium to repeat the process (Bachmann et al. 2013).

To ensure aerobic growth was supported, the oil was saturated with pressurized atmospheric air prior to use. Sufficient O_2_ supply was confirmed by measuring growth on a nonfermentable carbon source (data not shown).

### Growth Curve Measurements

Strains of interest were precultured and propagated to a 96-well plate. The spaces between wells were filled with 0.9% saline solution, and the plate sealed with parafilm to reduce evaporation from the wells. Growth was measured every 5 min, with shaking in between, overnight in a SpectraMax^®^ plate-reader, at 600 nm and 37 °C. Using the R software environment, growth rates, and maximum ODs were determined per strain as described earlier (Bachmann et al. 2013).

Alternatively, growth was measured manually. Strains were grown in 250 ml Erlenmeyer flasks, shaking at 220 RPM, at 37 °C. Every 30 min samples were taken to measure OD_600_ in cuvettes.

### Initial Screening after Evolution

After the experimental evolution 90 single colonies were randomly picked from the three parallel cell cultures and transferred to a 96-well plate containing 200 μl M9 + 2.5 mM glucose per well. Six wells were inoculated with the wild-type strain. After overnight growth, 20 µl from each well was transferred to a 96-well plates containing fresh medium for proper growth curve measurements. Glycerol was added to a final concentration of 12% and the plates were frozen at −80°C. Growth curves were analyzed for growth rates and maximal ODs. Several strains per replicate culture were selected for further analysis, based on the maximal OD compared with the wild-type.

### Cell Number and Volume Measurements

Cell number and volume were determined using the Coulter Counter^®^ Multisizer 3 (settings: aperture 30 µm; kd 41; current 800 µA; gain 16; sizing threshold 0.3 µm) after adding 50 μl cell culture to 10 ml ISOTON II electrolyte.

### Metabolic Product Measurements with HPLC

HPLC analysis was done on the (0.2 μm) filtrate of the culture supernatant to determine the metabolic products. The HPLC (Shimadzu LC20, 300x7.8 mm) with an ion exclusion column Rezex ROA Organic Acid H+ (8%), was run with 5 mM H_2_SO_4_, kept at 55 °C at a flow rate of 0.5 ml min^−1^. Calibrations were done with formate, lactate, acetate, orotate, and dihydroorotate.

### Glucose Determinations

Given the high concentration of phosphate in the M9 medium, and the overlap of this compound with glucose in the HPLC chromatogram, enzymatic glucose determinations were used to complete the metabolic profile. This was done by preparing a reaction mixture containing (final concentrations) PIPES pH = 7.0 (14.627 mM), NADP^+^ (0.286 mM), ATP (0.571 mM), MgSO_4_ (1.428 mM), hexokinase (30 U/ml), and glucose-6-phosphate dehydrogenase (35 U/ml). In a 96-well plate, 22 μl of sample or standard containing known concentrations of glucose were added to each well, then 178 μl of reaction mixture was added to every sample/standard. This was mixed, incubated for ∼30 min at 30°C, and measured at 340 nm until the curves were stationary. Glucose concentrations were calculated using the calibration curve (measured in duplicate), based on the final A_340_.

### Protein Content and Dry Weight

Total protein content of the selected strains was determined following the protocol of the Pierce^®^ BCA Protein Assay Kit. To prepare the samples for this assay, 2 ml of culture was transferred to an Eppendorf tube, washed in 0.9% saline solution, and the cell pellet was redissolved in 200 μl of saline. About 50 μl of SDS (10%) was added, and the samples were incubated at 90 °C for 1 h. Of these samples, and a set of calibration samples with known BSA concentrations, 25 μl was transferred to a 96-well plate and 200 μl of reagent (Pierce^®^ BCA Protein Assay Kit; Reagent A: Reagent B = 50:1) was added. Sample and reagent were mixed by shaking on a plate-shaker for 30 s, then incubated at 37 °C for 30 min. Protein content was subsequently determined in a SpectraMax^®^ plate-reader at 562 nm, and data were analyzed using the BSA-calibration curves (measured in duplicate), based on the final A_562_.

The dry weights of the selected strains were determined by filtering 50 ml of cell culture through a previously dried 0.2 μm pore nitrocellulose filter using a vacuum pump, drying these filters at 60°C to a constant weight and determining the difference in weight before and after the application of the samples to the filters, using a precision scale.

### Genome Resequencing

Genomic DNA was isolated using a Promega Wizard DNA purification kit. The quality of DNA was assessed with UV absorbance ratios by using a NanoDrop apparatus. DNA was quantified by using a Qubit dsDNA high-sensitivity assay. Paired-end resequencing libraries were generated using an Illumina Nextera XT kit with 1 ng of input DNA total. Sequences were obtained using an Illumina Miseq with a PE500v2 kit. The breseq pipeline (Deatherage and Barrick 2014) version 0.23 with bowtie2 was used to map sequencing reads and identify mutations relative to the *E. coli* K-12 substr. MG1655 genome (NCBI accession NC_000913.2). All samples had an average mapped coverage of at least 25.

### Model Description

The aim of this model is to evaluate the fitness of biphasic metabolism, incl. overflow metabolism, and compare it to the fitness of a pure-respiration strategy; to identify which parameters determine the winning metabolic strategy, that is, the one favored by evolution when cells are confronted with a finite amount of sugar that they can consume until its depletion.

We assume that the cells deplete a finite amount of sugar. This sugar amount limits the final number of produced cells, as all other nutrients are assumed in excess. Selection of cell number yield takes place when single cells are allowed to grow, while they are physically separated in emulsion droplets containing glucose-limited medium. In this scenario, the total time of growth does not matter. Selection of growth rate occurs in a well-mixed environment in a batch culture, also until all carbon sources have been depleted. In both cases, the winning evolutionary strategy made the most offspring after all carbon sources, that is, the limiting nutrients, have been depleted (sugar and overflow metabolites).

### Model Equations

The number of cells made from a finite starting amount of glucose, glc[0], when a fraction ϕ of the glucose uptake rate goes to acetate formation equals,

$$\underset{\mathrm{cells}}{\underset{︸}{\Delta N_{g}}}=\underset{\frac{\mathrm{cell}}{\mathrm{liter}}}{\underset{︸}{\frac{1}{V_{c,g}\;}}}\underset{\frac{\mathrm{liter}}{\mathrm{gram}}}{\underset{︸}{\frac{1}{\rho }}}\underset{\frac{\mathrm{gram}}{\mathrm{mol}}}{\underset{︸}{Y_{x/g}}}(1-\phi )\underset{\mathrm{mol}\;}{\underset{︸}{\mathrm{glc}[0]}}a\mathrm{nd}\;0\leq \phi \leq 1,$$

with the units of each factor explicitly indicated: $V_{c,g}$ = the volume of a single cell during growth on glucose, ρ = the mass density, and $Y_{x/g}$ = the gram biomass yield on glucose. Note that no cells are made when all glucose, during the glucose phase, is converted into acetate (i.e., ϕ=1). After the glucose growth period, with duration $T_{g}$, during which $\Delta N_{g}$ cells were made, the total number of cells equals,

$$N[T_{g}]=\Delta N_{g}+N[0]=e^{\mu_{g}[\phi ]T_{g}}N[0],$$

with N[0] as the starting number of cells, such that

$$\Delta N_{g}=\left(e^{\mu_{g}\left[\phi \right]T_{g}}-1\right)N\left[0\right]\;\mathrm{and}\;T_{g}=\frac{1}{\mu_{g}[\phi ]}\ln\left[\frac{\Delta N_{g}}{N[0]}+1\right].\;$$

The function $\mu_{g}[\phi ]$ denotes the growth rate during the glucose phase and is dependent on the fraction ϕ of glucose converted into acetate. Chemostat experiments indicate that fully respiring cells, with ϕ=0, grow slower than partially-overflowing cells with ϕ≈0.2. And, cells that only overflow, that is, ϕ=1, clearly do not grow on glucose at all. The dependency of the growth rate on glucose on ϕ, that is, $\mu_{g}[\phi ]$, therefore has to obey the following experimental observations:

1. when ϕ=0, it equals the growth rate at pure respiration, $\mu_{g}\left[0\right]=\alpha \mu_{a},$ which exceeds $\mu_{a}$ (the growth rate on acetate); thus, α>1, as a fully respiring cell growing on glucose grows faster than a cell growing on acetate (given chemostat and batch data).
2. at ϕ=1, the growth rate on glucose equals 0 as all glucose is converted into acetate.
3. a ϕ-value $\phi_{\mathrm{peak}}$ exists, 0 < $\phi_{\mathrm{peak}}<1,$ with a growth rate ${\mu_{g}(\phi }_{\mathrm{peak}})\;$that exceeds the growth rate at pure respiration $\alpha \mu_{a}$ (and $\mu_{a})$.

Thus, the function $\mu_{g}[\phi ]$ rises from a nonzero starting value $\mu_{g}\left[0\right]>\mu_{a}>0$ to a maximal value at $\mu_{g}\left[\phi_{\mathrm{peak}}\right]$ and then decreases again to 0 at $\mu_{g}[1]$. This behavior is an extrapolation of experimental data. The following function for the growth rate on glucose suits our purposes (with the Greek letters denoted positive parameters),

$$\mu_{g}\left[\phi \right]=\left(1-\phi \right)\left({\alpha +\delta \;e}^{-\frac{{\left(\beta -\phi \right)}^{2}}{\gamma^{2}}}\right),$$

which equals zero at ϕ=1 and is positive at ϕ=0 where its value equals $\alpha \mu_{a}.$ Note that this function shows the desired behavior, but is otherwise arbitrary.

The amount of acetate produced during the glucose growth period equals,

$$\underset{\mathrm{mol}\;\mathrm{acetate}}{\underset{︸}{\mathrm{ace}[T_{g}]}}=\underset{\frac{\mathrm{mol}\;\mathrm{acetate}}{\mathrm{mol}\;\mathrm{glucose}}}{\underset{︸}{Y_{a/g}}}\phi \underset{\mathrm{mol}\;}{\underset{︸}{\mathrm{glc}[0]}}.$$

The number of cells that we can make from this amount of acetate equals,

$$\underset{\mathrm{cells}}{\underset{︸}{\Delta N_{a}}}=\underset{\frac{\mathrm{cell}}{\mathrm{liter}}}{\underset{︸}{\frac{1}{V_{c,a}}}}\underset{\frac{\mathrm{liter}}{\mathrm{gram}}}{\underset{︸}{\frac{1}{\rho }}}\underset{\frac{\mathrm{gram}}{\mathrm{mol}}}{\underset{︸}{Y_{x/a}}}\underset{\mathrm{mol}\;\mathrm{acetate}}{\underset{︸}{\mathrm{ace}[T_{g}]}},$$

with $V_{c,a}$ as the volume of a cell during growth on acetate and the $Y_{x/a}$ as the gram biomass yield on acetate. Note that we neglect that from glucose-grown cells, more cells can be made than expected from the acetate yield and acetate amount, because glucose-grown cells are larger than acetate cells (see main text). This effect we neglect in the model, to keep it simple.

Thus, the number of cells at the end of the experiments, after glucose and acetate growth, equals

$$N\left[T_{a}+T_{g}\right]=\Delta N_{a}+N\left[T_{g}\right]\;\mathrm{and}\;N\left(T_{a}+T_{g}\right)=e^{\mu_{a}T_{a}}N\left[T_{g}\right],$$

with $T_{a}$ as the duration of the acetate growth period and $\mu_{a}$ as the growth rate on acetate. These last two equations allow us to determine the growth period on acetate,

$$T_{a}=\frac{1}{\mu_{a}}\ln\left[\frac{\Delta N_{a}}{N[T_{g}]}+1\right].$$

The total number of cells made from the starting amount of glucose equals,

$$N[T_{g}+T_{a}]=e^{\mu_{a}T_{a}}e^{\mu_{g}[\phi ]T_{g}}N[0].$$

The total number of cells made equals

$$\Delta N=N\left[T_{g}+T_{a}\right]-N[0].$$

We define the growth factor as

$$G[\phi ,V_{c,a},V_{c,g}]:=\frac{N[T_{g}+T_{a}]}{N[0]}{=e}^{\mu_{a}T_{a}}e^{\mu_{g}[\phi ]T_{g}}.$$

and the geometric fitness as

$$F[\phi ,V_{c,a},V_{c,g}]:=\frac{\ln G}{T_{g}+T_{a}}=\mu_{g}[\phi ]\frac{T_{g}}{T_{g}+T_{a}}+\mu_{a}\frac{T_{a}}{T_{g}+T_{a}},$$

which equals the time-averaged growth rate, since $\frac{T_{g}}{T_{g}+T_{a}}$ equals the fraction of time spent in phase 1 (glucose phase) and $\frac{T_{a}}{T_{g}+T_{a}}$ equals the fraction of time spent in the acetate phase. The $F[\phi ,V_{c,a},V_{c,g}]$ is plotted in figure 2*C*. See Table 1 for used parameter values.

**Table 1.** The Used Parameters for Figure  2.

Name	Value	Reference
Cell density	280 × 10^−15^ g/µm^3^	Milo and Phillips (2015)
Biomass yield on glucose	88 g/mol	Holms (1996)
Biomass yield on acetate	20 g/mol	Holms (1996)
Acetate yield on glucose	2 mol/mol	Holms (1996)
Cell volume on glucose (V_c,g_)	1 µm^3^	Si et al. (2017)
Cell volume on acetate (V_c,a_)	0.5 µm^3^	Si et al. (2017)
Growth rate on glucose (μ_g_)	1 h^−1^	Si et al. (2017)
Growth rate on acetate (µ_a_)	0.3 h^−1^	Si et al. (2017)
α	0.2	This study
β	0.5	This study
γ	0.4	This study
δ	2	This study

### A Complication: Cell Size and Growth Rate Are Related for *E. coli*

*Escherichia* *coli* has a fixed DNA replication time. This means that at long generation times (low growth rates) enough time exists between cell birth and division to replicate a single copy of DNA. This is not the case when the generation time is shorter than the DNA replication time: then DNA replication occurs continuously throughout the cell cycle and cells have started replication of DNA copies for future progeny. As a result, cells that grow fast have multiple origins of replication. It has been shown that the exponential relation between cell size and growth rate can be understood to result from the fact that *E. coli* maintains a constant ratio of cell size over the number of origins of replication. What matters for our model is that wild-type cells obey the following relation: $V(\mu )=V(0)e^{\varepsilon \mu }$ with $V(0)=0.28\;\mu m^{3}$ and ε=1.33 h (Si et al. 2017).

## Supplementary Material

msab345_Supplementary_Data
