# Supplementary material for: Selection for Cell Yield Does Not Reduce Overflow Metabolism in Escherichia coli
Source: Mol Biol Evol. 2021 Dec 6;39(1):msab345. doi: 10.1093/molbev/msab345 (PMC8789295; doi:10.1093/molbev/msab345)
Supplement: msab345_Supplementary_Data [file msab345_supplementary_data.pdf]

Supplementary Information

Selection for cell yield does not reduce overflow metabolism in *E. coli*

Iraes Rabbers[1], Willi Gottstein[1], Adam Feist[2], Bas Teusink[1], Frank Bruggeman[1], Herwig Bachmann[1,3]

[1]Systems Biology Lab, Vrije Universiteit Amsterdam, 1081 HV Amsterdam, The Netherlands,

[2] Department of Bioengineering, University of California, San Diego, La Jolla, CA, USA

[3] NIZO Food Research, Ede, The Netherlands

Keywords: overflow metabolism, r/k selection, yield, emulsion culturing, metabolic strategy, cell size

Correspondence: h.bachmann@vu.nl

Supplementary Information 1. Comparison of cell size under different growth conditions

An underlying assumption in the hypothesis above is that the cell size after entering stationary phase when growing on acetate is smaller than when entering stationary phase after growing on glucose. We found that when wildtype cells are growing in a glucose limited batch culture where the final divisions are on the overflow metabolite acetate, the cell size in stationary phase is approx. 55% smaller than the cell size during exponential growth on glucose. As strain MG1655 always produces acetate in a batch culture on glucose as sole carbon source, investigating the entering of stationary phase without acetate exposure in batch culture is not possible. To mimic the effects on cell size when going into stationary phase at a faster growth rate than on acetate we prepared nitrogen limited batch cultures where no biphasic growth is observed. The results showed that the final cell size after going into stationary phase with nitrogen limitation is approx. 24% smaller than during exponential growth (see Supplementary Figure 4), which is significantly bigger than cells entering stationary phase after growth on acetate.

A second assumption is that the growth rate reduction that comes with full respiration does not lead to a cell size decrease that combined with its effect on increasing biomass yield would lead to a higher cell yield than growth on acetate. We found 5 studies with *E. coli* where maximum growth rates and growth rates that still allow full respiration are reported (see Supplementary Table 4). All five show that a growth rate reduction to 77% - 59% of the maximum growth rate is sufficient for a strain to change metabolism towards full respiration. In our case the balanced growth rate on acetate is 28% and 31% of the maximum growth rate for MG1655 and IR1 respectively. This is therefore well below the growth rate reduction required to allow full respiration suggesting that the growth rate reduction on acetate as a substrate adds to the cell number through making smaller cells.

Supplementary Figures

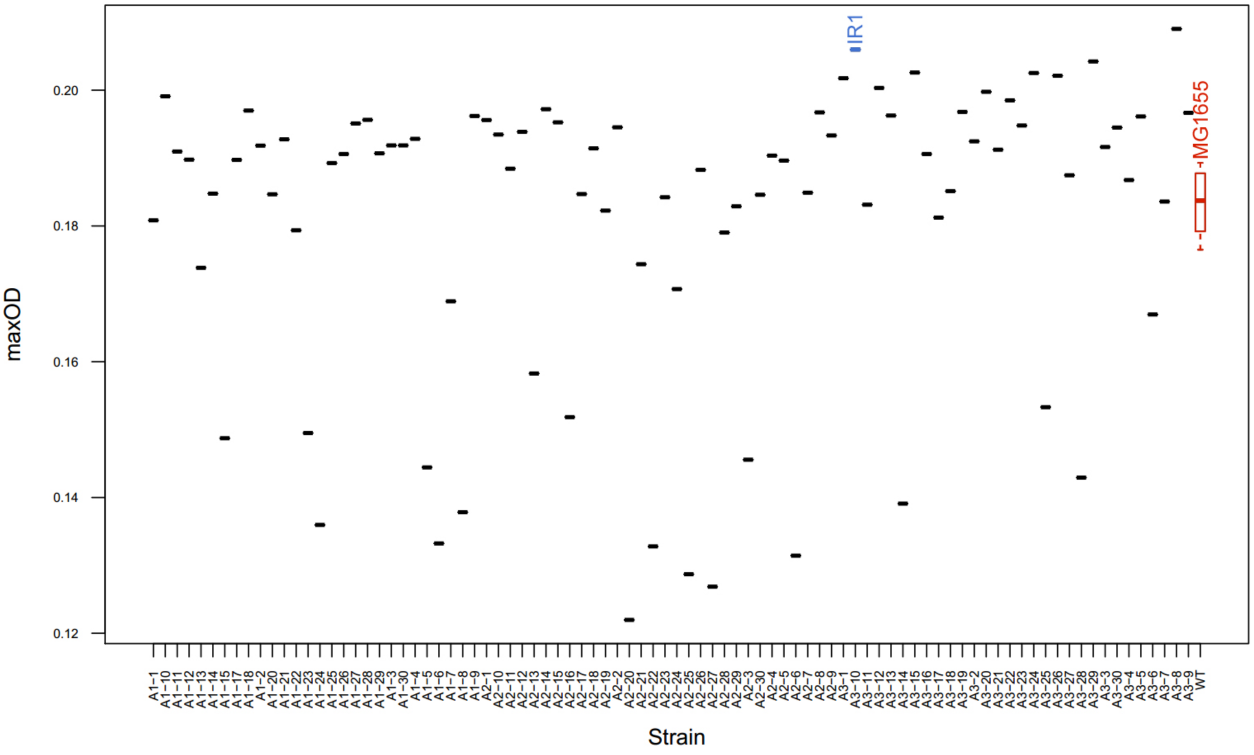

Supplementary Figure 1: After 60 (A1) or 53 (A2, A3) propagations in emulsion, three replicate populations A1, A2 and A3 were plated, and 90 single colonies were picked. The maximal optical densities at 600nm of these evolved strains were compared to that of the wildtype (boxplot of 6 replicates), to screen for strains with a suspected increase in yield. 5 strains were selected for extensive characterization (OD, dry weight, protein content, HPLC, Coulter Counter) based on the initial screening measurements, of which strain IR1 (colony 10 from population A3) is highlighted in this study.

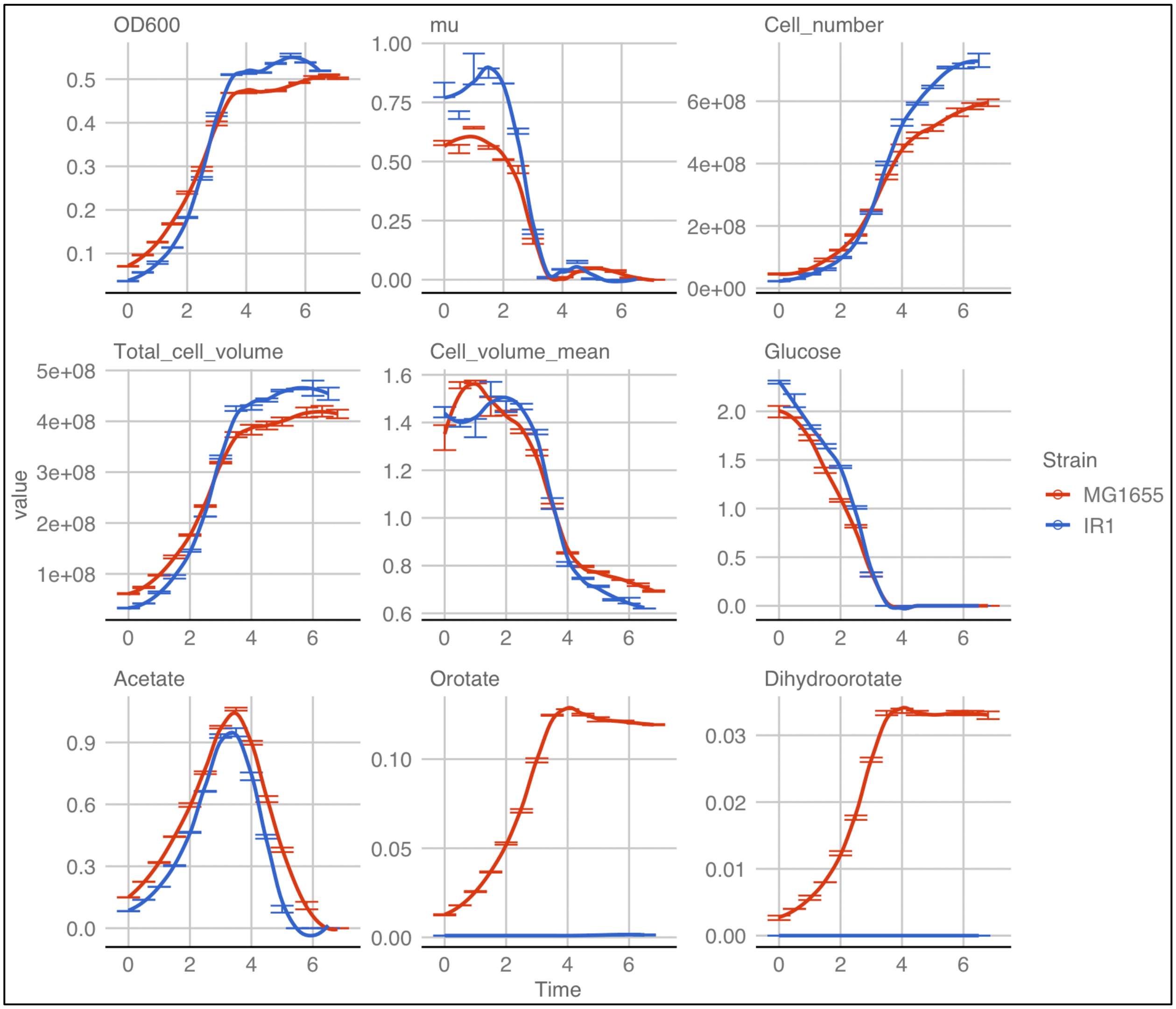

Supplementary Figure 2: Growth characteristics from batch cultures. Units: Growth rate mu [h^{-1}], Cell_number [number/ml culture^{-1}], Total_cell_volume [μm^3/ml culture], Cell_volume_mean [μm^3], Glucose [mM], Acetate [mM], Orotate [mM], Dihydroorotate [mM]. Error bars are standard errors of the mean, n=3. For completenes the panels from Fig 1 C-E (main text) are shown here as well.

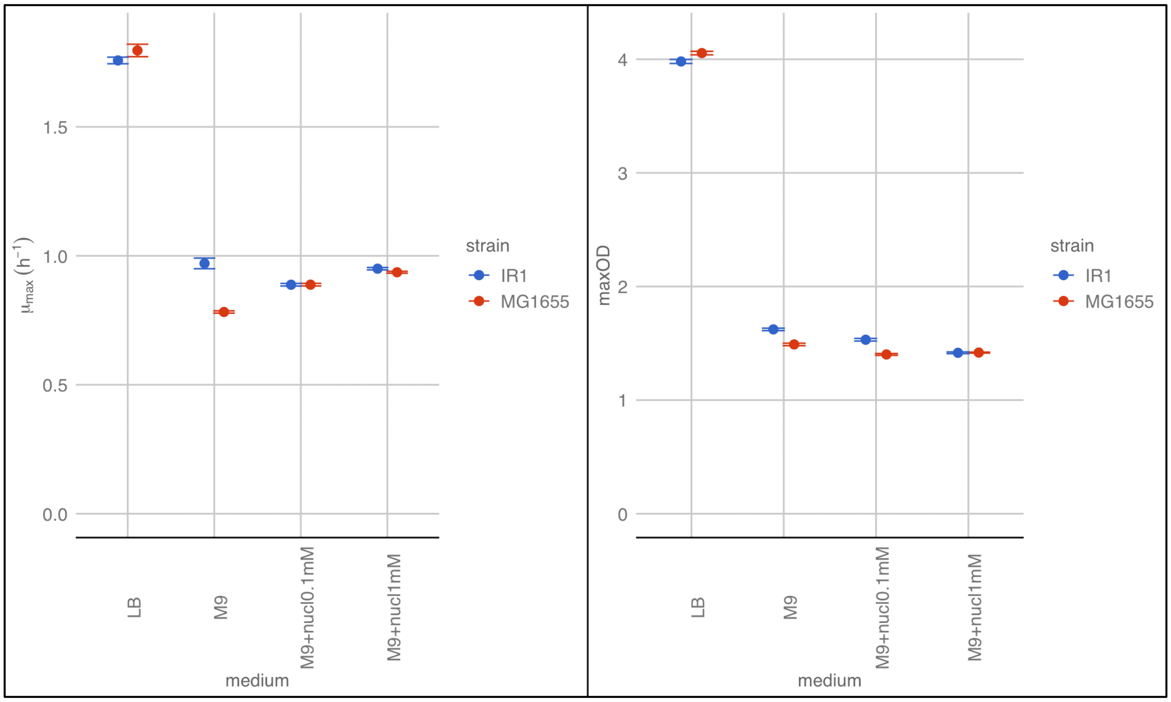

Supplementary Figure 3. Phenotypic properties of the wild type strain and IR1. Phenotypes associated with a mutation in the *rph-pyrE* region that strain IR1 acquired when evolved on M9 minimal medium, is consistent with an earlier described medium adaptation(Yates and Pardee 1956; Jensen 1993; Conrad et al. 2009; Valgepea et al. 2011). On rich LB medium the ancestral wildtype strain has a higher maximal specific growth rate (μ_{max}) and final optical density (maxOD) than strain IR1. When grown on M9 medium however, the pyrimidine production deficiency of the wildtype strain leads to a reduced μ_{max} and maxOD. If the minimal medium is supplemented with free nucleotides (0.1 or 1 mM), this disadvantage of the wildtype strain is alleviated. Means with SEM are shown, n=6.

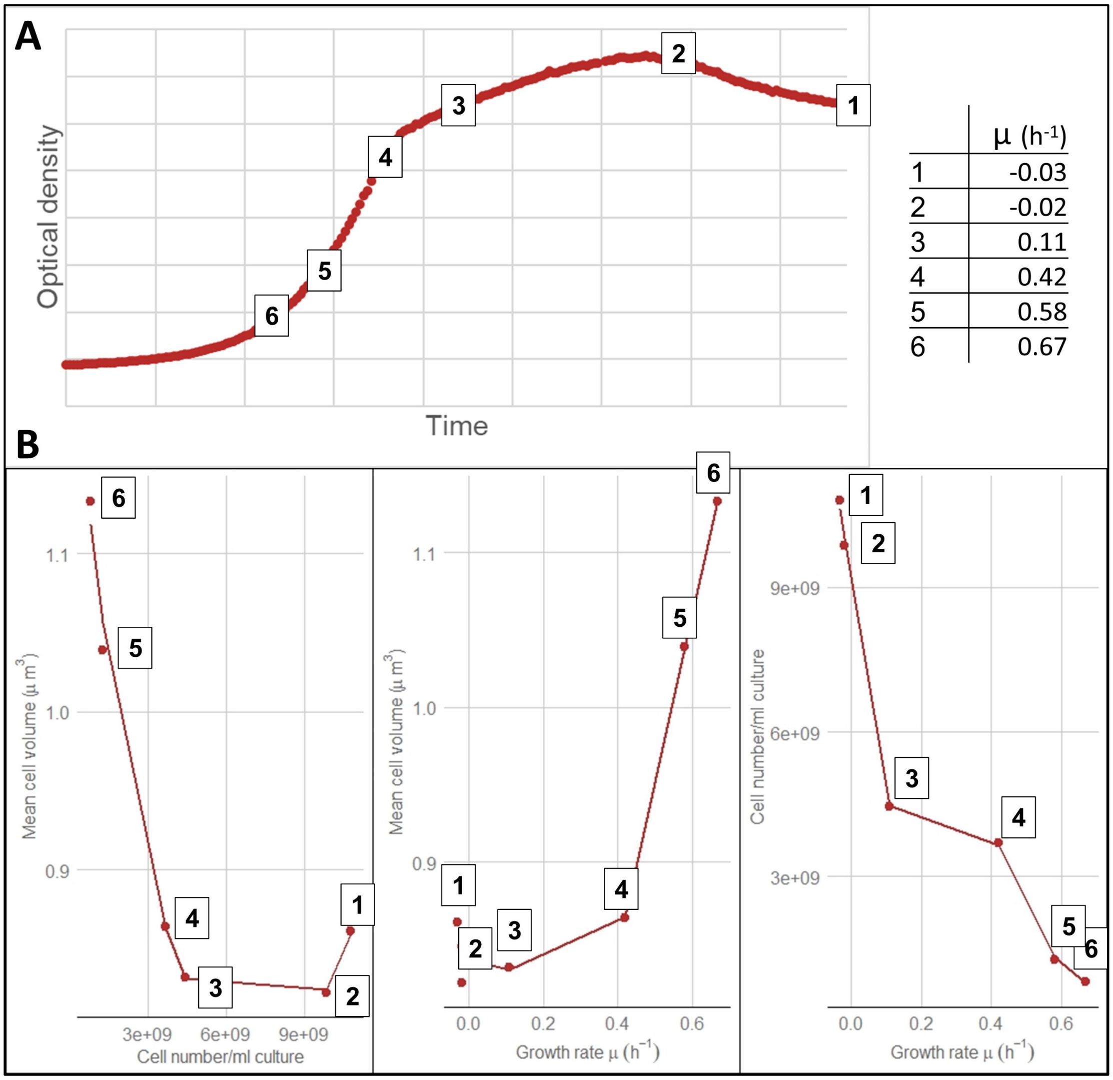

	μ (h^{-1})
1	-0.03
2	-0.02
3	0.11
4	0.42
5	0.58
6	0.67

Supplementary Figure 4: **A.** Wild type MG1655 was grown in batch on M9 supplemented with 50 mM glucose. In this case the growth medium is nitrogen limited instead of carbon limited hence cells still grow on glucose when going into stationary phase (no growth on acetate). Using a dilution series of different inoculation densities samples were taken at various timepoints throughout the growth curve for Coulter Counter measurements (i.e. cell volume and cell number). The growth rate (slope of log(OD)) was determined for different timepoints from mid-exponential to prolonged stationary phase. **B.** As the growth rate decreases, the cell volume also decreases while the cell number increases. In this nitrogen limited experiment, the decrease in cell volume from mid-exponential until stationary phase is around 24%. This decrease is significantly less than a decrease in cell volume of 55% observed for cells that become stationary in a glucose limited culture where a second growth phase on acetate occurs - see Supplemental Figure 2). Data was fitted with a smooth spline (smoothing parameter 0.3), n=2.

Supplementary Tables

Supplementary Table 1: Overview of the phenotypic characterization from wild type MG1655 and evolved strain IR1. Mean and standard deviation are given (n=3). Growth rates and maximal ODs were measured in shake flask (SF), as well as microplate (MP). All other measures come from the shake flask cultures. The second growth phase on acetate is too short to reach balanced growth, and sample handling for shake flasks can lead to some temperature reduction, which can cause a reduction in apparent growth rates. The growth rate difference between the wild type and evolved strain were therefore confirmed using microplate growth curves to ensure balanced growth conditions.

	**MG1655**		**IR1**		**IR1 % of**	**t-test (p-**
	**mean**	**st.dev.**	**mean**	**st.dev.**	**MG1655**	**value)**
cell number/ml culture	5.95E+08	1.93E+07	7.31E+08	3.77E+07	123	0.0117
glc exp cell volume (µm³)	1.57	0.01	1.54	0.05	98	0.4449
stationary cell volume (µm³)	0.70	0.01	0.62	0.00	89	0.0002
total cell volume/ml culture	4.14E+08	1.44E+07	4.54E+08	2.12E+07	110	0.0615
dry weight (mg/ml culture)	0.163	0.006	0.181	0.006	111	0.0208
total protein content (µg/ml culture)	306.0	8.5	345.4	10.2	113	0.0000
maximal OD_{600} - SF	0.510	0.002	0.555	0.006	109	0.0027
maximal specific growth rate glc (μ_{max} (h^{-1})) - SF	0.579	0.010	0.805	0.008	139	0.0000
maximal specific growth rate acet (μ_{max} (h^{-1})) during 2nd growth phase - SF	0.034	0.001	0.056	0.008	166	0.0359
maximal OD_{600} - MP	0.531	0.013	0.570	0.008	107	0.0154
maximal specific growth rate glc (μ_{max} (h^{-1})) - MP	0.839	0.015	1.100	0.005	131	0.0003
maximal specific growth rate acet (μ_{max} (h^{-1})) balanced growth - MP	0.224	0.007	0.294	0.011	131	0.0017
fraction of C to acetate (end of 1st growth phase)	0.159	0.005	0.150	0.003	94	0.0690
fraction of C to pyrimidine intermediates (end of 2nd growth phase)	0.051	0.001	0.000	0.000	1	0.0000

Supplementary Table 2: Strain IR1 was propagated for 25 additional cycles in emulsion, and 15 strains (3 per replicate evolution culture) were screened for acetate production after 3, 4 or 5 hours of batch growth. All strains still produced considerable amounts of acetate indicating that overflow metabolism was still present.

	**Acetate (mM)**			**OD600**			**Acetate/OD**		
**Strain**	**time=3h**	**time=4h**	**time=5h**	**time=3h**	**time=4h**	**time=5h**	**time=3h**	**time=4h**	**time=5h**
A1-C2	0.549	0.946		0.157	0.370	0.513	3.50	2.56	
A1-F5	0.528	0.912		0.152	0.323	0.509	3.47	2.82	
A1-D5	0.554	0.969		0.158	0.347	0.507	3.51	2.79	
A1-F6	0.537	0.934		0.154	0.320	0.507	3.49	2.92	
A1-E6	0.536	0.959		0.156	0.339	0.509	3.44	2.83	
A2-B6	0.736	1.237		0.223	0.528	0.530	3.30	2.34	
A2-D5	0.725	1.327		0.205	0.495	0.506	3.54	2.68	
A2-E9	0.648	1.258		0.196	0.464	0.490	3.31	2.71	
A2-E6		0.847	1.278	0.105	0.264	0.514		3.21	2.49
A2-G7	0.745	1.316		0.210	0.503	0.522	3.55	2.62	
A3-E7	0.568	1.03		0.173	0.370	0.505	3.28	2.78	
A3-G11	0.594	1.099		0.177	0.401	0.495	3.36	2.74	
A3-D12	0.695	1.313		0.213	0.465	0.492	3.26	2.82	
A3-B12	0.86	1.247		0.249	0.530	0.523	3.45	2.35	
A3-G12		0.791	1.286	0.095	0.241	0.482		3.28	2.67
MG1655	0.441	0.733	1.091	0.114	0.198	0.327	3.87	3.70	3.34
IR1	0.572	1.068		0.176	0.378	0.505	3.25	2.83	

Supplementary Table 3: Mutations identified in the genome of strain IR1.

**position**	**mutation**	**annotation**	**gene**	**description**
2,760,454	C→A	intergenic (+60 / -93)	*yfjI* → / → *yfjJ*	CP4-57 prophage; uncharacterized protein/CP4-57 prophage; uncharacterized protein
2,999,264	G→A	Q210* (CAA → TAA)	*ygeR* ←	LytM domain-containing M23 family putative peptidase; septation lipoprotein
3,026,033	Δ1 bp	coding (268 / 1320 nt)	*guaD* →	guanine deaminase
3,815,859	Δ82 bp	pseudogene (610-691 / 716 nt)	*rph* ← *pyrE*	ribonuclease PH (defective); enzyme; degradation of RNA; RNase PH
3,984,420	T→A	T530S (ACC→ TCC)	*aslA* ←	putative Ser-type periplasmic non-aryl sulfatase

Supplementary Table 4: Identifying growth rates of *E. coli* which allow full respiration. The two rows at the bottom of the table show that for the two strains used in this study the growth rate reduction on acetate is much bigger compared to the growth rate reduction needed for cells to fully respire (top part of the table).

**Reference**	**Figure/ table**	**Strain**	**Culture method**	**Medium**	**μ at which cells still show full respiration**	**μ_{max} /highest D used in paper**	**Fraction of μ_{max} at which cells still show full respiration**
Nanchen (2006)(Nanchen et al. 2006)(Nanchen et al. 2006)	Fig. 3	MG1655 (l- F- rph-1Fnr+; DSMZ)	chemostat	M9 minimal	0.4	0.7	0.57
Holms (1996)(Holms 1996)(Holms 1996)	Table 7	ML308 = ATCC15224	chemostat		0.72	0.94	0.77
Valgepea (2010)(Valgepea et al. 2010)(Valgepea et al. 2010)	Fig. 2	K12 MG1655 (l- F- rph-1Fnr+; DSMZ)	chemostat	defined minimal	0.27	0.46	0.59
Renilla (2012)	Fig 4	BW25113 a K12-derivative	chemostat	defined minimal	0.5	0.7	0.71
(Meyer et al. 1984)	Fig. 1	defined medium	chemostat		0.35	0.54	0.65
(Basan et al. 2015)	Fig. 1	K12=MG1655	batch	minimal	0.76	1.26	0.60

				**μ_{max} on acetate**	**μ_{max} on glucose**	**Fraction of μ_{max}**
This Study	MG1655	batch	M9 minimal	0.22	0.84	0.26
This Study	IR1 - evolved	batch	M9 minimal	0.29	1.1	0.26

Supplementary Table 5: Growth rates on acetate and glucose, and the ratio between them for a number of different organisms and strains. [a]Strain not able to grow on glucose, therefore malate was taken as a reference high quality carbon source. [b]Strain unable to grow on glucose, so no ratio was calculated.

**Organism**	**μ on acetate**	**μ on glc**	**μ_{ac}/μ_{glc}**	**Reference**
E. coli MG1655	0.22	0.84	0.28	This study
E. coli IR1	0.29	1.10	0.31	This study
Yarrowia lipolytica	0.28	0.41	0.68	(Robak 2007)
Corynebacterium glutamicum	0.28	0.32	0.88	(Wendisch et al. 2000)
Rhodobacter capsulatus	0.29	0.42[a]	0.71	(Willison 1988)
E. coli B/r strain NF790	0.30	0.89	0.34	(Andersen and von Meyenburg 1980)
S. cerevisiae AG1-7	0.12	0.31	0.39	(Johnston et al. 1979)
E. coli BW25113	0.29	0.60	0.48	(Volkmer and Heinemann 2011)
Neurospora crassa	0.41	0.51	0.80	(Alberghina et al. 1975)
Acinetobacter schindleri[b]	0.91			(Sigala et al. 2019)

References

Alberghina FA, Sturani E, Gohlke JR. 1975. Levels and rates of synthesis of ribosomal ribonucleic acid, transfer ribonucleic acid, and protein in Neurospora crassa in different steady states of growth. *J. Biol. Chem.* 250:4381–4388.

Andersen KB, von Meyenburg K. 1980. Are growth rates of Escherichia coli in batch cultures limited by respiration? *J. Bacteriol.* 144:114–123.

Conrad TM, Joyce AR, Applebee MK, Barrett CL, Xie B, Gao Y, Palsson BT. 2009. Whole-genome resequencing of Escherichia coli K-12 MG1655 undergoing short-term laboratory evolution in lactate minimal media reveals flexible selection of adaptive mutations. *Genome Biol.* 10.

Holms H. 1996. Flux analysis and control of the central metabolic pathways in Escherichia coli. *FEMS Microbiol. Rev.* 19:85–116.

Jensen KF. 1993. The Escherichia coli K-12 “wild types” W3110 and MG1655 have an rph frameshift mutation that leads to pyrimidine starvation due to low pyrE expression levels. *J. Bacteriol.* 175:3401–3407.

Johnston GC, Ehrhardt CW, Lorincz A, Carter BL. 1979. Regulation of cell size in the yeast Saccharomyces cerevisiae. *J. Bacteriol.* 137:1–5.

Nanchen A, Schicker A, Sauer U. 2006. Nonlinear dependency of intracellular fluxes on growth rate in miniaturized continuous cultures of Escherichia coli. *Appl. Environ. Microbiol.* 72:1164–1172.

Robak M. 2007. Yarrowia lipolytica specific growth rate on acetate medium supplemented with glucose, glycerol or ethanol. *Biotechnologia* 6:23–31.

Sigala J-C, Quiroz L, Arteaga E, Olivares R, Lara AR, Martinez A. 2019. Physiological and transcriptional comparison of acetate catabolism between Acinetobacter schindleri ACE and Escherichia coli JM101. *FEMS Microbiol. Lett.* 366.

Valgepea K, Adamberg K, Nahku R, Lahtvee PJ, Arike L, Vilu R. 2010. Systems biology approach reveals that overflow metabolism of acetate in Escherichia coli is triggered by carbon catabolite repression of acetyl-CoA synthetase. *BMC Syst. Biol.* 4.

Valgepea K, Adamberg K, Vilu R. 2011. Decrease of energy spilling in Escherichia coli continuous cultures with rising specific growth rate and carbon wasting. *BMC Syst. Biol.* 5:106.

Volkmer B, Heinemann M. 2011. Condition-Dependent Cell Volume and Concentration of Escherichia coli to Facilitate Data Conversion for Systems Biology Modeling. 6:1–6.

Wendisch VF, de Graaf AA, Sahm H, Eikmanns BJ. 2000. Quantitative determination of metabolic fluxes during coutilization of two carbon sources: comparative analyses with Corynebacterium glutamicum during growth on acetate and/or glucose. *J. Bacteriol.* 182:3088–3096.

Willison JC. 1988. Pyruvate and Acetate Metabolism in the Photosynthetic Bacterium Rhodobacter capsulatus. *Microbiology* 134:2429–2439.

Yates RA, Pardee AB. 1956. Control of pyrimidine biosynthesis in Escherichia coli by a feed-back mechanism. *J. Biol. Chem.* [Internet] 221:757–770. Available from:

https://linkinghub.elsevier.com/retrieve/pii/S0021925818651889
